# Soil Sickness in Aged Tea Plantation Is Associated With a Shift in Microbial Communities as a Result of Plant Polyphenol Accumulation in the Tea Gardens

**DOI:** 10.3389/fpls.2020.00601

**Published:** 2020-05-28

**Authors:** Yasir Arafat, Israr Ud Din, Muhammad Tayyab, Yuhang Jiang, Ting Chen, Zhaoying Cai, Hanyu Zhao, Xiangmin Lin, Wenxiong Lin, Sheng Lin

**Affiliations:** ^1^Key Laboratory of Fujian Province for Agroecological Process and Safety Monitoring, College of Life Science, Fujian Agriculture and Forestry University, Fuzhou, China; ^2^Key Laboratory for Genetics, Breeding and Multiple Utilization of Crops, Ministry of Education, College of Crop Sciences, Fujian Agriculture and Forestry University, Fuzhou, China; ^3^Institute of Biotechnology and Genetic Engineering, The University of Agriculture Peshawar, Peshawar, Pakistan

**Keywords:** indirect allelopathy, catechins, soil sickness, monoculture problems, plant polyphenol

## Abstract

In conventional tea plantations, a large amount of pruned material returns to the soil surface, putting a high quantity of polyphenols into the soil. The accumulation of active allelochemicals in the tea rhizosphere and subsequent shift in beneficial microbes may be the cause of acidification, soil sickness, and regeneration problem, which may be attributed to hindrance of plant growth, development, and low yield in long-term monoculture tea plantation. However, the role of pruning leaf litter in soil sickness under consecutive tea monoculture is unclear. Here, we investigated soil samples taken from conventional tea gardens of different ages (2, 15, and 30 years) and under the effect of regular pruning. Different approaches including liquid chromatography–mass spectrometry (LC-MS) analysis of the leaf litter, metagenomic study of root-associated bacterial communities, and *in vitro* interaction of polyphenols with selected bacteria were applied to understand the effect of leaf litter-derived polyphenols on the composition and structure of the tea rhizosphere microbial community. Our results indicated that each pruning practice returns a large amount of leaf litter to each tea garden. LC-MS results showed that leaf litter leads to the accumulation of various allelochemicals in the tea rhizosphere, including *epigallocatechin gallate*, *epigallocatechin*, *epicatechin gallate*, *catechin*, and *epicatechin* with increasing age of the tea plantation. Meanwhile, in the tea garden grown consecutively for 30 years (30-Y), the phenol oxidase and peroxidase activities increased significantly. Pyrosequencing identified *Burkholderia* and *Pseudomonas* as the dominant genera, while plant growth-promoting bacteria, especially *Bacillus*, *Prevotella*, and *Sphingomonas*, were significantly reduced in the long-term tea plantation. The qPCR results of 30-Y soil confirmed that the copy numbers of bacterial genes per gram of the rhizosphere soil were significantly reduced, while that of *Pseudomonas* increased significantly. *In vitro* study showed that the growth of catechin-degrading bacteria (e.g., *Pseudomonas*) increased and plant-promoting bacteria (e.g., *Bacillus*) decreased significantly with increasing concentration of these allelochemicals. Furthermore, *in vitro* interaction showed a 0.36-fold decrease in the pH of the broth after 72 h with the catechin degradation. In summary, the increase of *Pseudomonas* and *Burkholderia* in the 30-Y garden was found to be associated with the accumulation of catechin substrates. In response to the long-term monoculture of tea, the variable soil pH along with the litter distribution negatively affect the population of plant growth-promoting bacteria (e.g., *Sphingomonas*, *Bacillus*, and *Prevotella*). Current research suggests that the removal of pruned branches from tea gardens can prevent soil sickness and may lead to sustainable tea production.

## Introduction

Plant soil feedbacks can alter the composition and structure of the soil microbial community and nutrient homeostasis as a result of all the interactions between plants and soil organisms, affecting soil fertility and plant growth (Kaur et al., 2009; Bever et al., 2010; van de Voorde et al., 2012; Baxendale et al., 2014). Soil disease or replanting disease is plant-soil negative feedback caused by consecutive planting of a single crop or its related species on the same field, resulting in a reduction in crop yield and quality (Muller, 1966; Huang et al., 2013; Zhao et al., 2015, 2016; Wu L. et al., 2016). Long-term monoculture not only impedes the growth and production of many annual crops, trees, and shrubs in orchards but also causes replanting and regeneration problems (Canals et al., 2005). Therefore, it is pertinent to understand the mechanisms underlying soil sickness associated with long-term monoculture practice, to explore the allelochemical interaction with soil microbiota, and to provide a solution for maintaining a sustainable agro-ecosystem.

Degradation of autotoxins or allelochemicals by microorganisms has been reported in previous studies (Bever et al., 2010; Weidenhamer et al., 2013). Several cinnamic and benzoic acids were initially detected in the Cecil Ap horizon soil, and after a few days, they were not detected in amended soil (Blum et al., 2000). Phenolics, being the most abundant plant metabolites, are thought to control the rates of soil organic matter decomposition and can be applied as a tool to evaluate the soil dynamics and ecosystem functioning (Min et al., 2015). Hence, the effects of soil microbiota are critical to the fate of plant phenolic compounds, including other potential allelochemicals found in the soil (Bever et al., 2010; Ehlers, 2011).

Polyphenols enter the soil in the form of leachates from above plant parts as plant residues and litter (Hättenschwiler and Vitousek, 2000). Contributions related to these input pathways, mainly underground flows, have a limited understanding. In most of the less disturbed terrestrial ecosystems, plant growth and net primary productivity depend on recycled nutrient availability, while external nutrient inputs contribute little to the total requirement. The rate-limiting steps in the nutrient cycle, such as climate, substrate quality (litter), and decomposing microbes, are decisive at influencing soil microbial mineralization (Heal, 1997). Polyphenols influence soil microbial activity and soil physicochemical properties, indicating interactions with nutrient cycling, thereby modifying the flux and pool of soil nutrients available to microbes and/or plants (Hättenschwiler and Vitousek, 2000). Several studies suggest that through specific mechanisms, soil macrofauna increases the biodegradation and mineralization of soil organic matter (Wardle and Lavelle, 1997) and high concentrations of polyphenols could limit such fauna in terms of activity and abundance (Neuhauser, 1978). Consequently, the direct effect of polyphenols on soil fauna is hard to explain owing to the complexity of soil food webs and the co-variability of other compounds.

Tea is an important economic crop, widely cultivated in Southeast Asia and China (Chen and Lin, 2015) and contains several phenolic compounds throughout the tea plant, especially in the leaves, accounting for 18–34% of the dry weight of the leaves (Wan, 2003). In tea plantation systems, the conventional management approach, especially pruning, is employed twice a year in terms of keeping tea bushes in the best shape, improving tea quality, increasing yield, and even inhibiting diseases and pests (Yilmaz et al., 2004; Maudu et al., 2010). The trimmed tea leaves and branches are rich in nutrients and are often amended into tea gardens to improve the soil physicochemical properties, soil organic matter, and nutrient availability (Weeraratna et al., 1977). However, some studies have demonstrated that the decomposition of accumulated residues and litter produces allelochemicals, mainly phenolic compounds, flavonoid, and alkaloids, which, in turn, inhibit the microbial activities in the rhizospheric soil. The indirect allelopathy of pruned tea leaves, especially polyphenols on soil sickness in long-term tea cropping systems, needs further consideration. The objectives of this study were to inspect the impact of tea litter and its polyphenols on (a) root-related bacterial communities in terms of structure and composition, (b) ratooning or regeneration problems related to soil sickness, and (c) growth and quality parameters of tea plants in response to continuous tea cultivation. Assuming that polyphenols represent a possible allelochemical substance, they regulate the tea soil bacterial community feedback processes, which leads to the progressive imbalance during long-term tea monocropping. In addition, changes in microbial community structure and composition play an important role in soil sickness as compared to the direct allelopathy.

## Materials and Methods

### Site Overview

Current experiment on tea plantation was carried out at Fujian Agriculture and Forestry University tea fields, Fuzhou City, Fujian Province, China (latitude: 26°0509.60′′ N; longitude: 119°1403.60′′ E) (27°43′ N, 118°72′ E). The area has a subtropical monsoon climate, with average annual temperature and rainfall (20–25°C, and around 900–1362 mm, respectively).

### Soil Sampling

Rhizosphere soil along with roots was taken from different aged tea gardens (2, 15, and 30 years) and bulk soil (CK) from the same fields was used as control. The location, agronomic management practices, and environmental conditions of the tea gardens were the same. To overcome error began by spatial heterogeneity, five randomly taken soil samples from 15 sampling locations were combined into one replication, and three replications were carried out for each sample. In order to deliver soil samples to the laboratory, all soil samples were immediately stored in a sterile icebox (Lin et al., 2013). For the analysis of soil enzymes and microbes, a part of each soil sample and roots were stored at −80°C, and the remainder was air-dried for physicochemical attributes. With little modification in the method proposed by Edwards et al. (2015), we determined the microbial fauna in 2-Y, 30-Y, and CK soil. Shortly, the bacterial communities were obtained from tea root compartments, e.g., RS, rhizosphere (soil firmly adhered to the root surface); RP, rhizoplane (suite of microbes present on the root surface by sonication); and ES, endosphere (interior of the same plant roots after sonication).

### Quality Parameters, Growth Index, and Yield Determination

Theophylline (TPY), theanine (TNN), and the total polyphenol (TPP) content of the uppermost leaves taken from three plants grown in each tea garden, i.e., 2-Y, 15-Y, and 30-Y, was determined by the method proposed by Peng et al. (2008) for quality determination. To examine the total chlorophyll content of the third leaf, eight plants were randomly selected from each garden and values were recorded using an instrument (SPAD-502 Plus). Further, using the US portable CO_2_ gas analyzer (CID-301), the net photosynthetic rate (Pn) of the third leaf was measured. Three replicates for each sample were taken to perform Pn. One hundred buds’ weight (fresh and dry) was estimated in grams for each of three biological replications.

### Soil Enzyme Activities Analysis

With a slight modification, the activity of soil polyphenol oxidase and peroxidase was determined by the method previously adopted (Perucci et al., 2000; Saiya-Cork et al., 2002; Xu et al., 2015). Briefly, 1 g of soil was added to a 100-ml flask containing 10 ml of 1% pyrogallol before incubation at 300°C for 2 h, and then 35 ml of ether was added into the flask and then shaken for 15 min on a thermostat oscillator at 250°C. Moreover, peroxidase activity was determined using the same procedure along with the addition of 2 ml of a 0.5% H_2_O_2_ solution, and the final absorbance was recorded at 430 nm.

### Leaf Litter Biomass Determination and Identification and Quantification of Allelochemicals

#### Standard Materials and Their Calibration Curves

In order to quantify and identify concentration of allelochemicals in leaf litter samples, ≥98% pure catechin, epigallocatechin (EGC), epicatechin gallate (ECG), epicatechin (EC), epigallocatechin gallate (EGCG), taxifolin (TF), and protocatechuic acid (PCA) were purchased as external standards from Cayman Chemical (1180 E. Ellsworth Road Ann Arbor, MI, United States). Formic acid and LC-MS grade methanol were also purchased from Sigma-Aldrich (St. Louis, MO, United States). For a stock solution preparation, 1 mg of every standard was suspended in 1 ml of 99.9% methanol and 0.1% by volume of formic acid. Serial dilutions were made using the same solvent as the stock solution, with a dilution range of 1.25–10 μg/ml in order to obtain a calibration curve. Each solution was distinctly injected with a 10-μl aliquot for HPLC-ESI-MS analysis. With all correlation coefficients > 0.998, excellent linearity was achieved within the calibration range (Supplementary Figures S1, S2).

#### Allelochemicals Determination From Leaf Litters

The leaf litter biomass that comes on the soil surface from each tea plant per pruning was determined in grams (Supplementary Figure S3). Leaf litters were collected randomly from three tea plants in each tea garden and were weighed by a digital balance. By adopting the method of Wang et al. (2013) with slight modifications, the allelochemicals concentration in leaf litter was determined by HPLC-ESI-MS. We took 5 g of the litter and fermented it for 3 days in 15 ml of a solution (containing 50% methanol and 0.1% acetic acid) at room temperature (25°C), and after three times sonication, it was vortexed at 150 rpm for 24 h. By vaporization at ultra-pressure, the methanol extract was air-dried and the crude extract was passed through a C-18 column (open column). After centrifugation at 4500 rpm for 10 min, the supernatant was then shifted to a sample collection vessel for liquid chromatography (LC).

#### The Chromatographic Conditions for Allelochemical Determination

Following this, HPLC–ESI–MS was carried out using a T3 RP-18 column (100 × 2.1 mm; 5 μm; Waters, Milford, MA, United States), eluted with buffer A (0.1% acetic acid) and buffer B (100% methanol) at a flow rate of 300 μl/min at 25°C. Initially, the column was eluted with 95% buffer B, followed by a linear increase in buffer A to 35% from 0 to 10 min, and further maintained in 90% buffer A until 10.50 min. Then, a linear increase in buffer B to 95% was maintained. Finally, the column was maintained in 95% buffer B for up to 19 min. The total time for running one sample was 19 min. The negative ionization mode was selected to perform mass spectrometry at a temperature of 100°C, and ion scans were carried out at low-energy collision (20 eV) using nitrogen as the collision gas. All the data from HPLC-ESI-MS were processed to determine the mean concentrations of the selected catechin compounds in each sample, by using Bruker Daltonics Data analysis software (version 4.0).

### The Metagenomic Analysis of the Root-Associated Bacterial Communities

#### Soil DNA Extraction

Fresh soil samples were passed through a 2-mm mesh sieve and then 0.5 g was taken in 2-ml Eppendorf tubes for DNA extraction. DNA was extracted using a BioFast Soil Genomic DNA Extraction Kit (Bioer Technology Co., Ltd., Hanzhou, China) according to the manufacturer’s instructions. In order to estimate the quality and concentration of soil DNA, NanoDrop 2000 spectrophotometer (Thermo Scientific, Waltham, MA, United States) was used. DNA was diluted to 1 ng/μl using sterile water accordingly to concentration.

#### PCR Amplification

Primers 515F/806R with the barcode were used in order to amplify the distinct region of 16S rRNA gene (16S V4). All PCR reactions were conducted with Phusion^®^ High-Fidelity PCR Master Mix (New England Biolabs). PCR conditions were (95°C for 3 min, followed by 35 cycles of 95°C for 30 s, 55°C for 30 s, and 72°C for 45 s, with a final extension at 72°C for 10 min) (GeneAmp 9700, ABI, United States). PCR reactions were carried out in triplicate in 20-μl mixture having 2 μl of 2.5 mM deoxyribonucleoside triphosphate (dNTPs), 4 μl of 5 × Fast Pfu buffer, 0.4 μl of Fast Pfu polymerase, 0.4 μl of every primer (5 μM), and template DNA (10 ng) (TransGen catalogue no. AP221–02). PCR products were purified with Qiagen Gel Extraction Kit (Qiagen, Germany).

#### Library Preparation and Illumina Hi Sequencing

Purified PCR products were sent to Novogene Bioinformatics Technology Co., Ltd., (Beijing, China) for high-throughput sequencing. Sequencing libraries were generated using TruSeq^®^ DNA PCR-Free Sample Preparation Kit (Illumina, United States) following manufacturer’s recommendations and index codes were added. The library quality was assessed on the Qubit@ 2.0 Fluorometer (Thermo Scientific) and Agilent Bioanalyzer 2100 system. At last, the library was sequenced on an IlluminaHiSeq2500 platform and 250-bp paired-end reads were generated.

### Processing and Analyzing of Data

#### Paired-End Reads Assembly and Quality Control

Paired-end reads was assigned to samples based on their unique barcode and truncated by cutting off the barcode and primer sequence. Paired-end reads were merged using FLASH (V1.2.7)^1^ (Magoč and Salzberg, 2011), and the splicing sequences were called raw tags. Quality filtering on the raw tags were performed under specific filtering conditions to obtain the high-quality clean tags (Bokulich et al., 2013), according to the QIIME(V1.7.0)^2^ (Caporaso et al., 2010). The tags were compared with the reference database(Unite Database)^3^ using UCHIME algorithm (UCHIME Algorithm^4^) (Edgar et al., 2011) to detect chimera sequences, and then the chimera sequences were removed (Haas et al., 2011).

#### OTU Cluster and Species Annotation

Sequence analysis was performed by Uparse software (Uparse v7.0.1001)^5^ (Edgar, 2013). Sequences with ≥97% similarity were assigned to the same OTUs. Representative sequence for each OTU was screened for further annotation. For each representative sequence, the Unite Database^6^ (Kõljalg et al., 2013). was used basing on Blast algorithm, which was calculated by QIIME software (Version 1.7.0)^7^ to annotate taxonomic information.

#### Heat Map and Graph Construction

Heat map was preceded by ggplot2 package in R software (Version 2.15.3).

### Quantitative PCR Analysis of Total Bacteria in Ratooning Monoculture Tea Soil

A quantitative PCR (qPCR) assay was conducted to determine the size of bacterial population in the rhizosphere soil of different aged tea plantations. The Universal primer set Eub338/Eub518 was used to estimate the bacterial community size. qPCR was performed according to the method of Wu H. et al. (2016).

### qPCR Analysis of *Pseudomonas* and *Bacillus* Population in Ratooning Monoculture Tea Soil

A qPCR assay using the primer sets Psf/Psr (Chen et al., 2017) was also conducted to determine the copy number of *Pseudomonas* in tea rhizosphere soil after different years of monoculture. However, for *Bacillus* genus, we used the method of Wu H. et al. (2016). The 15-μl PCR reaction contained 7.5 μl of 2 × SYBR green I Super Real Premix (TransGen, Beijing, China), 0.5 μl of each primer (10 μM), 1 μl of 20 ng/μl template DNA, and 5.5 μl of RNase-free H_2_O. Serial dilutions of plasmid DNA were set as standard curve.

### *In vitro* Interactions of Allelochemicals With Selected Model Bacteria

#### Preparation of LB Medium

LB powder, 5 g, was mixed in 200 ml of ultrapure water in a 250-ml Erlenmeyer flask, covered the top with aluminum foil, and autoclaved at 121°C for 15 min.

For LB agar plates preparation, 3 g (1.5%) of bacteriological agar was added to 200 ml of LB liquid medium (5 g broth powder in 200 ml of ddH_2_O) in a 2500 ml Erlenmeyer flask and autoclaved at 121°C for 15 min. After autoclaving, when medium was cooled to ∼50°C, 200 μl of pimaricin antifungal (0.06 g/1 ml methanol) was added to 200 ml of media. The LB agar media was poured into the petri plates.

#### Preparation of Soil Serial Dilution

Five grams of soil was added to 45 ml of ddH_2_O in a 50-ml Erlenmeyer flask. The soil samples were diluted up to 10^–3^ times. Soil suspension (60 μl) of 10^–2^ and 10^–3^ dilutions was subjected to each hard-nutrient agar plate surface. The soil suspension was spread until it was equally distributed on hard agar plate. Last, the plates were incubated at 30°C for 24–72 h, and the colonies that show dark zone surrounding them were separated from a mixed bacterial community in the culture.

#### Sub-Culturing of Bacteria

Already prepared LB agar plates were used to get each purified bacterial colony. For this, divide the plates under sides by drawing equal numbers of squares to each plate and label it by giving specific numbers and then pick each colony carefully by sterilizing the pipette tip and attaching it to each label square. The plates were incubated at 30°C for 24–72 h again. These colonies represent each bacterial strain.

#### Enrichment and Preservation of Bacteria

The sterilized LB liquid broth was prepared and 5-ml broth solutions were distributed in sterilized 5-ml tubes. After that, one colony into each tube was suspended and incubated at 30°C for 72 h. To preserve each bacterium, put 1 ml of bacterial suspension in a sterilized Eppendorf tube and add an equal amount of glycerol (1:1) to it and preserve these colonies at −20°C for further use. DNA was extracted from each labeled bacterial suspension. The DNA were sent to the company for 16s RNA sequencing to identify key microbes.

#### The Influence of Identified Allelochemicals on the Growth of Selected Model Microbes

The LB Liquid broth was diluted six times and 5 ml was distributed in each glass tube; all tubes were sterilized at 121°C for 20 min. After autoclaving, the broth was amended with an appropriate amount of identified allelochemical stock solution that was filtered through a 0.22-μm filtration membrane. The final concentrations were 0, 1.25, 2.5, 5, and 10 μg/ml, respectively, for each allelochemical. The control was set in the same amount of ddH_2_O instead of allelochemical. Each treatment was repeated three times. Fifty microliters of each labeled bacteria was added to each tube, which has been already activated. All tubes were placed in a thermostatic shaker at 30°C and 200 rpm for 72 h. Further, 200 μl of bacterial culture was taken in a 96-hole enzyme label plate (Thermo Scientific Multiskan Mk3, Shanghai, China). To obtain the appropriate bacterial growth, the OD at 600 nm was regularly checked (Wu H. et al., 2016).

#### Biotransformation of Catechin and Its Effect on Soil pH

The LB liquid broth was diluted six times to minimize LB nutrient effect on bacterial growth and 5 ml was distributed in each glass tube. All the tubes were sterilized at 121°C for 20 min. After precooling, 50 μl of each labeled bacteria was added to each tube that has been already activated. The broth was amended with an appropriate amount of EC stock solution that was filtered through a 0.22-μm filtration membrane. After growing bacteria, an appropriate amount of broth was centrifuged at 13,000 rpm for 5 min at 4°C. The supernatant was taken for pH determination and for identification of their metabolites by LC-ESI-MS at 24, 48, 72, 96, and 120 h, respectively (Wang et al., 2013).

### Statistical Analysis

All the experiments included three replications and repeated at least two times. The effects of the different treatments were analyzed by LSD test using SPSS 19.0, and the graphs were displayed using GraphPad Prism 5 software.

## Results

### The Performance of Tea Plants in Consecutively Ratooned Monoculture Soil

**FIGURE 1:**
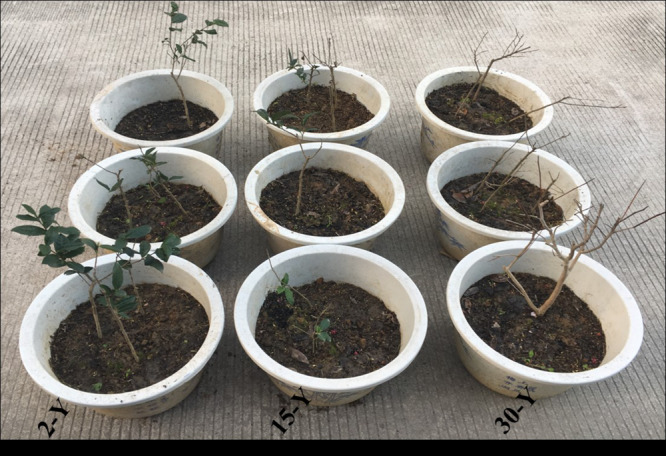
Replanting problems in continuous monoculture tea garden soil. 2-Y, 15-Y, and 30-Y indicate tea garden soil in which tea was planted continuously for different years (2, 15, and 30 years).

**FIGURE 2:**
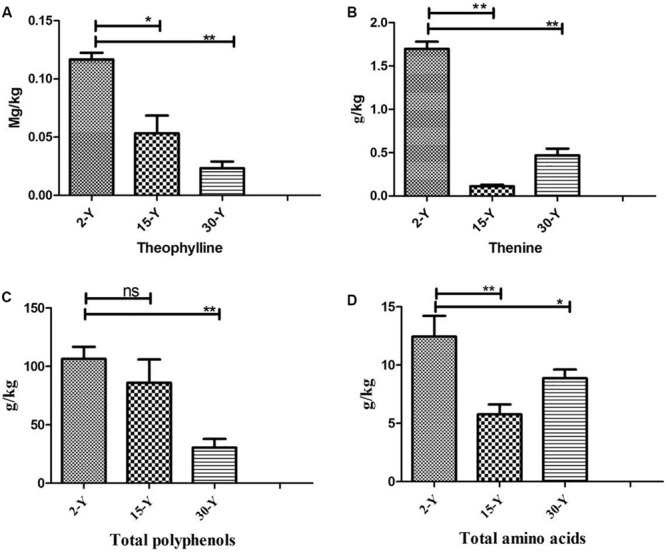
Quality parameters of tea leaves from tea plantations of different ages. **(A)** Theophylline, **(B)** theanine, **(C)** total polyphenols, and **(D)** total free amino acids. Stars in the column show significant differences (LSD test, *P* < 0.05, *n* = 3). 2-Y, 15-Y, and 30-Y indicate tea gardens planted continuously for different years (2, 15, and 30).

**FIGURE 3:**
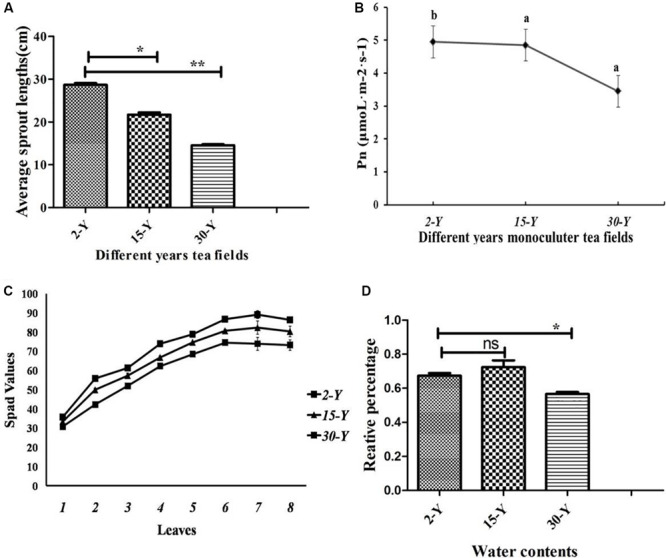
Physiological characteristics of tea leaves. **(A)** Length of new tea sprouts (*P* < 0.05, *n* = 5), **(B)** photosynthetic rate (Pn) of the third tea leaf in young sprout (*P* < 0.05, *n* = 3), **(C)** content of tea leaf chlorophyll with young sprouts from bottom to top (*P* < 0.05, *n* = 8), **(D)** water contents in five leaves. Stars in the column show significant differences (LSD test, *P* < 0.05, *n* = 5) under different fields. 2-Y, 15-Y, and 30-Y indicate tea gardens planted continuously for different years (2, 15, and 30).

Tea plants grown in soil taken from different tea plantations (15-Y and 30-Y) showed weak growth, wilting, chlorosis, and regeneration obstacles compared to tea plants grown in the recently established tea garden (2-Y) soil (Figure 1). In addition, compared to the 2-Y garden, tea quality parameters, especially theophylline (TPY), total polyphenols (TPP), theanine (TNN), and total amino acids (TAA), were significantly lower in old gardens (15-Y and 30-Y) as shown (Figures 2A–D). Regarding physiological and growth parameters, continuous tea cropping significantly reduced net photosynthetic rate (Pn), new tea sprout’s length, and chlorophyll content of the third leaf (Figures 3A–D). Supplementary Table S1 shows the tea yield obtained from the tea gardens with different planting histories. In comparison with 2-Y gardens, the old gardens (15-Y and 30-Y) significantly reduced 100 buds fresh and dry weight (Supplementary Table S1). These results indicate that tea quality and productivity are negatively affected by long-term tea monoculture.

### Analysis of Soil Enzyme Activities in Different Age Monoculture Tea Plantations

The activity of phenol oxidase peroxidase were promoted by 13.78, 14.92, and 51.52% in 2-, 15-, and 30-Y tea plantations, respectively, as compared to CK. Likewise, the activity of peroxidase was sharply increased by 11.58, 10.12, and 17.88%, respectively, in 15- and 30-Y tea plantations as compared to CK (Supplementary Table S2).

### Leaf Litter Biomass Input Into Tea Gardens and Analysis of Their Allelochemicals

**FIGURE 4:**
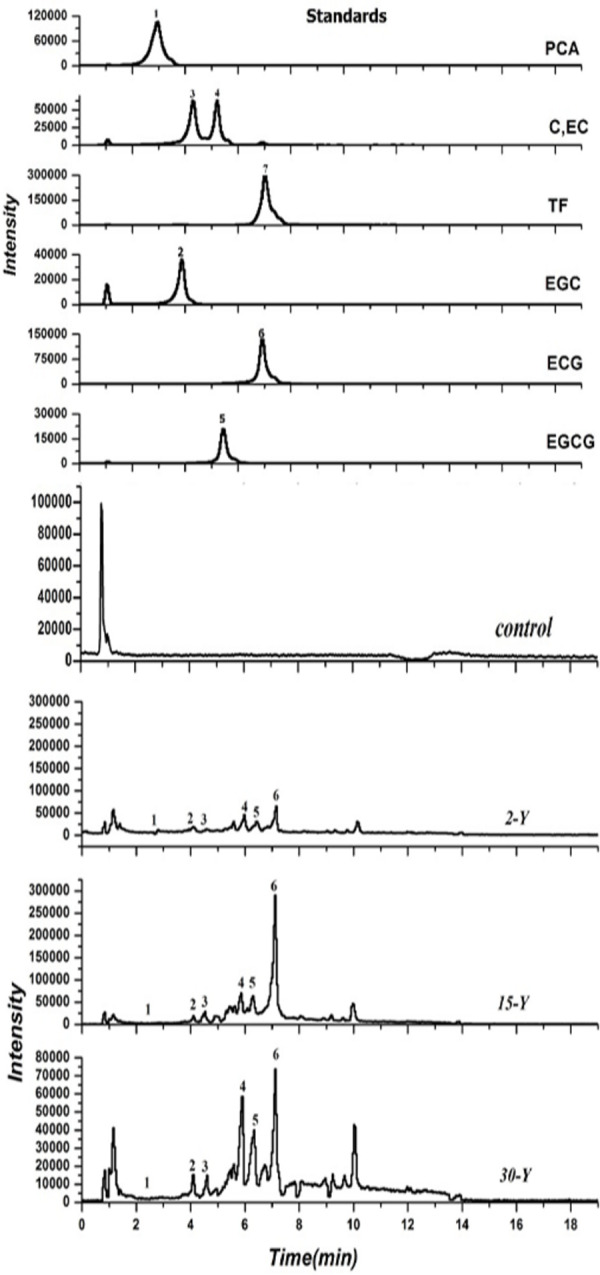
HPLC–ESI–MS spectra of catechins in leaf litters collected. (1) Represents protocatechuic acid (PCA) with a retention time of 3.08–3.12 min; (2) represents epigallocatechin (EGC) with a retention time of 4.07–4.11 min; (3) is catechin (±C) with a retention time of 4.52–4.55 min; (4) is epicatechin (EC) with a retention time of 5.42–5.44 min; (5) represents epigallocatechin-3-gallate (EGCG) with a retention time of 5.62–5.72 min; (6) represents epicatechin-3-gallate (ECG) with a retention time of 7.11–7.13 min; and (7) represents taxifolin (TF) with a retention time of 7.32–7.35 min. Control, 2-Y, 15-Y, and 30-Y indicate tea garden leaf litters planted continuously for different years (0, 2, 15, and 30).

Our results indicated that in each pruning turn, about 615, 838, and 969.67 g/plant leaf litters were returns into 2-Y, 15-Y, and 30-Y garden, respectively (Supplementary Figure S3). Five compounds including EGC, EGCG, EC, catechin (±C), and ECG, were identified in the leaf litters in both the newly planted tea gardens (2-Y) and the continuously monocultured tea gardens (15-Y and 30-Y) (Figure 4). Moreover, in the continuous monoculture tea gardens (15-Y and 30-Y tea gardens) the input of EGC, ±C, EC, EGCG, and ECG allelochemicals were high as compared to young tea plantations (2-Y).

### Response of Bacterial Genera Under Continuous Monoculture Soil

**FIGURE 5:**
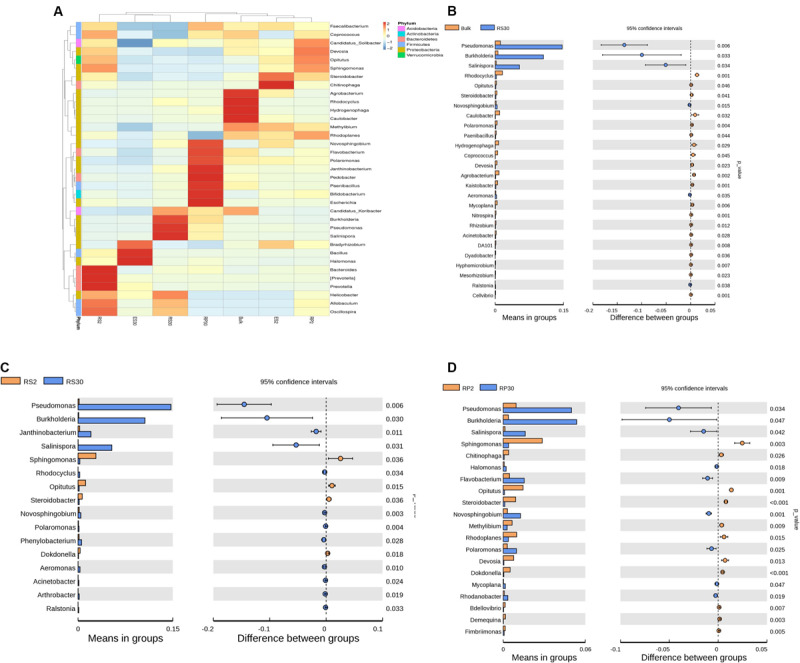
**(A)** Heat map showing the distribution of the 35 most abundant genera. Bulk soil (CK), rhizosphere (RS2 and RS30), rhizoplane (RP2 and RP30), and endosphere (ES2 and ES30) of tea gardens continuously cropped for 2 and 30 years, respectively (LSD test, *P* < 0.05, *n* = 3). **(B)** Error bar plots displaying significantly difference of most abundant genera among in Bulk (CK) and 30-Y rhizosphere (RS30) tea plantation (*t*-test, *P* < 0.05, *n* = 3). **(C)** Error bar plots displaying significant difference of most abundant genera in the 2-Y rhizosphere (RS2) and 30-Y rhizosphere (RS30) tea plantation (*t*-test, *P* < 0.05, *n* = 3). **(D)** Error bar plots displaying significant difference of most abundant genera in 2-Y rhizoplane (RP2) and 30-Y rhizoplane (RP30) tea plantation (*t*-test, *P* < 0.05, *n* = 3). The points explain differences among (“CK and RS30”), (“RS2 and RS30”), and (“RP2 and RP30”) (red, green, and blue bars, respectively); the values on the right-hand side display the *P*-values derived from the *t*-test error bar plots.

Heat map analysis displayed that *Acidobacteria (Candidatus_koribacter)* and *Proteobacteria* (*Rhodocyclus, Agrobacterium, Caulobacter*, *Hydrogenophaga*, and *Methylibium*) were the dominant bacterial phyla in CK (Figure 5A). The genera (e.g., *Pseudomonas, Burkholderia, Salinospora*, and *Helicobacter*) were dominant in RS30, whereas *Devosia, Coprococcus*, *Opitutus*, *Sphingomonas, Prevotella, Allobaculum, Bacteroides*, and *Oscillospira* were dominant in RS2. In RP2, the dominant genera included *Candidatus_solibacter, Opitutus, Devosia, Sphingomonas*, and *Rhodoplanes*, while in RP30, the dominant groups found were of *Flavobacterium*, *Novosphingobium*, *Polaromone*, *Pedobacter*, *Janthinobacterium*, *Paenibacillus*, *Escherichia*, and *Bifidobacterium*, respectively. Similarly, *Chitinophaga*, *Steroidobacter*, *Bradyrhizobium*, and *Methylibium* were predominant in ES2, and *Bradyrhizobium*, *Halomonas*, and *Bacillus* were predominant in ES30. Stamp analysis verified that *Salinispora*, *Pseudomonas*, and *Burkholderia* were dominant genera in both rhizoplane (RP30) and rhizosphere (RS30) compartments of old tea garden (30-Y) as compared with Bulk soil (CK) and fresh soil tea plantation (2-Y) rhizosphere (RS2) and rhizoplane (RP2) (Figures 5B–D, respectively).

### Abundance of Total Bacteria and *Pseudomonas* and *Bacillus* Genera by Colony-Forming Units’ qPCR Methods

**FIGURE 6:**
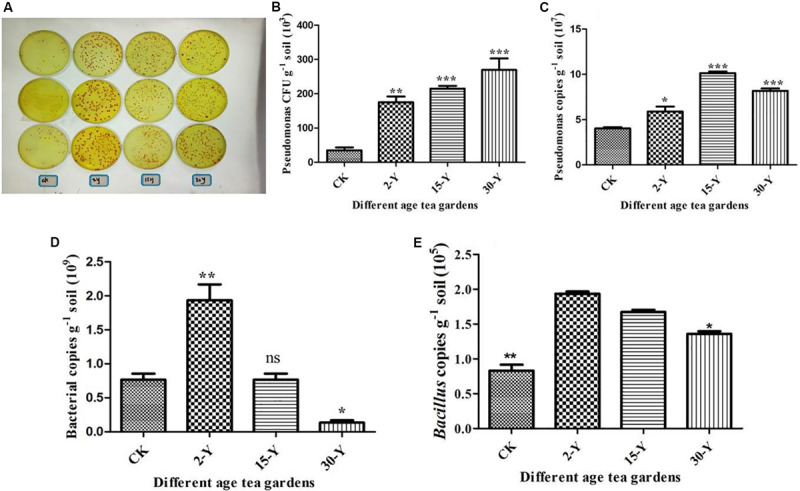
Abundance of total bacteria, *Pseudomonas* and *Bacillus* genera by qPCR analysis. **(A)** *Pseudomonas* populations. **(B)** CFU of *Pseudomonas* per gram of soil. **(C)** The contents of *Pseudomonas* genera in tea rhizosphere soils after different years of monoculture by qPCR analysis using the primer sets Psf/Psr (Tan and Ji, 2010). **(D)** The total bacterial contents by qPCR analysis using the primer set Eub338/Eub518. **(E)** The contents of *Bacillus* genera in tea rhizosphere soils after different years of monoculture by qPCR analysis (Wu H. et al., 2016). CK, 2Y, 15Y, and 30Y refer to bulk soil without planting any crop, newly planted 2-year garden, and replanted 15- and 30-year garden, respectively. Stars in the column show significant differences (LSD test, *P* < 0.05, *n* = 3).

Employing high-throughput sequencing, colony-forming units, and qPCR, we characterized the entire bacterial community composition and structure, including *Pseudomonas* and *Bacillus* in the CK, 2-Y, and 30-Y tea gardens. These results indicated that within 30-Y, the entire bacterial population and plant growth-promoting bacterial genus (*Bacillus*) were reduced compared to 2-Y and CK soil, while the catechin degradation bacteria (*Pseudomonas*) was increased (Figure 6).

### *In vitro* Interactions of Different Types of Allelochemicals With Model Growth-Promoting Bacteria

**FIGURE 7:**
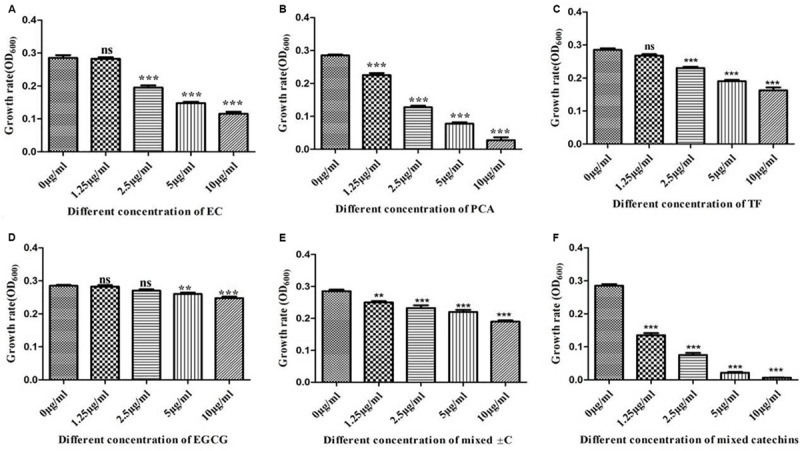
*In vitro* interactions of different types of allelochemicals with model growth-promoting bacteria *Bacillus.* **(A)** Epicatechin (EC), **(B)** protocatechuic acid (PCA), **(C)** taxifolin (TF), **(D)** epigallocatechin-3-gallate (EGCG), **(E)** catechin (±C), and **(F)** mix of all catechins. Stars in the column show significant differences (LSD test, *P* < 0.05, *n* = 4).

Based on the HPLC-LC/MS identification of different types of active catechins allelochemicals in the leaf litter, a series of single and mixed allelochemicals with the final concentration gradients (0, 1.25, 2.5, 5, and 10 μg/ml) were set to identify the response of selected model growth-promoting bacteria such as *Bacillus* found in the tea garden. Our results determined that the different allelochemicals concentration significantly influenced the growth of selected growth-promoting bacteria. Moreover, as the concentration of these identified allelochemicals increased, the growth of *Bacillus* decreased (Figure 7).

### *In vitro* Interactions of Different Types of Allelochemicals With Model Catechins Degrading Bacteria

**FIGURE 8:**
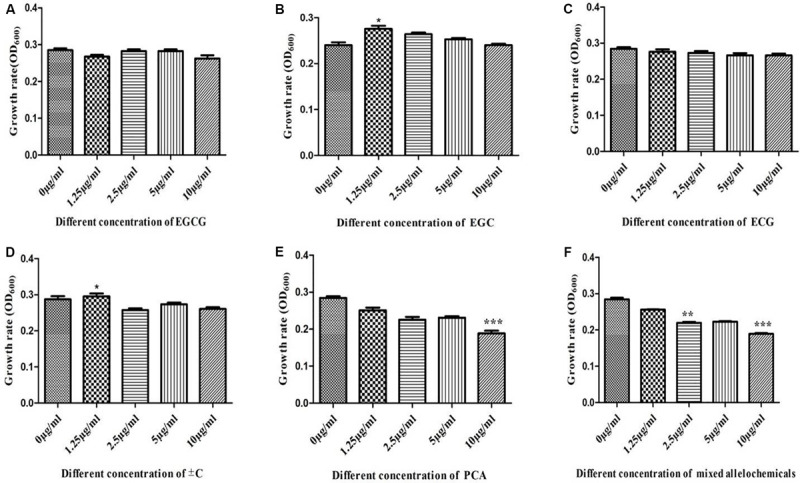
*In vitro* interactions of different types of allelochemicals with model catechins degrading bacteria *Pseudomonas.* **(A)** Epigallocatechin-3-gallate (EGCG), **(B)** epigallocatechin (EGC), **(C)** epicatechingallate (ECG), **(D)** catechin (±C), **(E)** protocatechuic acid (PCA), and **(F)** mix of all catechins. Stars in the column show significant differences (LSD test, *P* < 0.05, *n* = 4).

Similarly, a series of single and mixed allelochemicals with the final concentration gradients (0, 1.25, 2.5, 5, and 10 μg/ml) were also set to identify the response of catechin degrading bacteria, *Pseudomonas* spp., which was dominant in the 30-Y consecutively monoculture tea garden. The results showed that the different allelochemicals concentration have no significant effect on the growth of *Pseudomonas* spp. Low concentration at 1.25 μg/ml of ±C promoted the growth of *Pseudomonas* spp. However, the high concentration (10 μg/ml) of PCA and a mix of seven known allelochemicals significantly inhibit the growth of the selected model bacteria. It is therefore suggested that the dominant bacteria such as *Pseudomonas* used these leaf litters as a carbon substrate to some extent (Figure 8).

### Biotransformation of Catechins and Their Effect on pH

**FIGURE 9:**
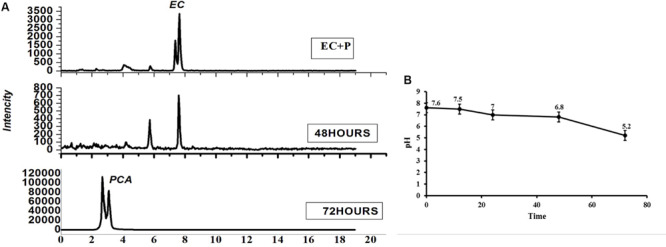
Impact of catechin degradation on pH. **(A)** Catechin degradation, **(B)** effect on pH. EC + P represents Epicatechin (EC) + *Pseudomonas* (*P*) and PCA represents protocatechuic acid.

In order to understand the role of the dominant bacterial populations in lowering the pH of the tea rhizosphere with the increasing tea planting age and consecutive monoculture problems, the dominant *Pseudomonas* was selected as a model bacterium. Epicatechin (EC) with the final concentration gradients 5 μg/ml and activated *Pseudomonas* bacterium (50 μl) was subjected to six times diluted LB medium. After 48 and 72 h, the media were purified and filtered for pH and metabolite analysis. The LC-MS analysis results identified the compound that was PCA after 72 h (Figure 9A). Moreover, results showed that the pH of the media drops to 0.36-fold after 72 h (Figure 9B). These results suggested that the dominant bacteria identified in 30-Y tea plantation used leaf litter. After using these substrates, the bacteria convert these allelochemicals into different types of acids like PCA, which may change the pH of the tea rhizosphere soil over time.

## Discussion

Our results indicated that long-term monoculture has an adverse effect on tea quality (TNN, TPY, TPP, and TAA content) and physiological and growth parameters of tea plants compared to freshly grown tea plants, which is consistent with previous findings (Arafat et al., 2017; Jiang et al., 2019). Similarly, previous studies have shown that tea production, as well as quality, declines dramatically with increasing cultivation time (Illukpitiya et al., 2004; Arafat et al., 2019). Owing to shifts in soil physicochemical and biological properties, a long-term tea monoculture can lead to a “soil-sickness” or “replanting disease,” which has an adverse impact on tea productivity (Owuor, 1996; Huang et al., 2006; Utkhede, 2006; Kamau, 2007; Qu and Wang, 2008). The underlying factors of soil sickness are usually considered to be nutrient imbalances, autotoxins generation, and/or shift in soil microbial community structure and diversity (Zhao et al., 2015). Plant-associated microbial communities are also considered as a second genome of the plant and are essential for soil fertility and plant health (Berendsen et al., 2012). The regulation of soil microbiota owing to allelochemicals interaction is closely associated with replanting disease in agricultural systems (Li et al., 2014; Liu et al., 2017).

Soil microbial biomass is commonly considered to be associated with soil fertility, including as a potential indicator of soil quality (Shengchun and Yinghua, 2003; Zhang and Zhang, 2003). Due to changes in soil management approaches, soil microbial dynamics shift faster than soil organic matter (Shengchun and Yinghua, 2003). Beneficial microbes, especially plant growth-promoting bacteria, are crucial for nutrient availability and plant growth (Acosta-Martínez et al., 2010; Cipollini et al., 2012; Huang et al., 2013). In the present study, we found *Burkholderia* and *Pseudomonas* as the dominant genera, while plant growth-promoting bacteria such as *Prevotella*, *Bacillus*, and *Sphingomonas* were significantly lower in the 30-Y tea garden. The qPCR results also confirmed that the bacterial density per gram of soil of 30-Y plantation was significantly lower compared to 2-Y. However, *Pseudomonas* population was selectively increased in 30-Y. It is generally believed that tea garden soils have an inhibitory effect on soil microorganisms due to high acidity and aluminum toxicity. We have previously reported that continuous planting of tea (30 years) has no significant effect on soil nutrients (N, P, and K). However, soil pH in continuous tea fields (30 years) was significantly lower than in the bulk and 2-year garden soil (2-Y) (Arafat et al., 2017).

Various effects of plant polyphenols have previously been observed under artificial laboratory conditions, indicating that they stimulate or inhibit (depending on the compound) the growth of the nitrifiers (Rice and Pancholy, 1973; Baldwin et al., 1983). Schimel et al. (1998), proving that plant polyphenols have a controlling effect on nutrient dynamics and species interaction in Alaskan taiga. The complexity of soil food webs, including the covariance of other compounds, makes it challenging to demonstrate the effects of polyphenols on soil biota directly. HPLC-LC/MS findings and *in vitro* interaction of the selected model growth-promoting bacteria with allelochemicals suggest that the various types of active catechins’ allelochemical concentration as they enhanced, in turn, significantly suppressed *Bacillus* growth (Figure 7). *In vitro* analysis of soil microbial density by catechin showed inhibition and microbe suppression (Inderjit et al., 2009). Catechin-degrading bacteria (*Pseudomonas* spp.) were greater in terms of abundance in the 30-Y tea garden and different concentrations of allelochemicals did not significantly influence the growth of *Pseudomonas* spp. (Figure 8). At a lower concentration (1.25 μg/ml of ±C), the growth of *Pseudomonas* was increased; on the other hand, a high frequency (10 μg/ml) of PCA and the combination of seven known allelochemicals significantly suppressed selected bacteria growth. These results suggest that dominant bacteria such as *Pseudomonas* utilized these catechins in leaf litter as a carbon substrate to a certain level. Wang et al. (2013) also showed that *Pseudomonas* can use catechins as substrate and biotransformed it into most toxic allelochemicals. We aimed to examine if the dominant bacterial groups associated with tea roots in the 30-Y tea garden has a role in soil acidification. EC with the final concentration of 5 μg/ml, which can activate the growth of *Pseudomonas*, was investigated for biotransformation and resulting metabolites. By LC-MS analysis, we detected compounds, especially PCA, and reduced the pH of the media (0.36 folds) after 72 h, indicating that abundant bacteria detected in the 30-Y garden converted the polyphenols in different types of acids (PCA, ferulic acids, etc.), which may lead to soil acidification.

## Conclusion

Overall, long-term tea monocropping had a significant impact on the tea in terms of both quality and quantity, as well as soil regenerative capacity. We also studied the polyphenol interaction with microorganisms in tea residue and rhizosphere, which ultimately declined the plant growth-promoting bacteria. However, below a particular concentration gradient, no significant effect was observed on the growth of *Pseudomonas*. We found that *Pseudomonas* biotransformed polyphenols in various organic acids (PCA, TF, and EC) and changed soil pH, which may be the putative reason for altering the microbial structure and composition of tea rhizosphere during continuous tea planting. We recommend eliminating plant residues and trimmed materials from the tea plantation to avoid the accumulation of allelochemicals, which can delay soil acidification.

## Data Availability Statement

The data set has been submitted to the NCBI-Sequence Read Archive with the SRA accession: PRJNA626070. Temporary Submission ID: SUB7295720.

## Author Contributions

WL, SL, and YA conceived the study. YA and IU wrote the manuscript. YA, YJ, and ZC performed field sampling and lab experiments. YA, IU, TC, and HZ performed the statistical analyses. All authors discussed the results and commented on the manuscript.

## Conflict of Interest

The authors declare that the research was conducted in the absence of any commercial or financial relationships that could be construed as a potential conflict of interest.

## Footnotes

1 http://ccb.jhu.edu/software/FLASH/

2 http://qiime.org/index.html

3 https://unite.ut.ee/

4 http://www.drive5.com/usearch/manual/uchime_algo.html

5 http://drive5.com/uparse/

6 https://unite.ut.ee/

7 http://qiime.org/scripts/assign_taxonomy.html
