# Supplementary material for: Soil Sickness in Aged Tea Plantation Is Associated With a Shift in Microbial Communities as a Result of Plant Polyphenol Accumulation in the Tea Gardens
Source: Front Plant Sci. 2020 May 28;11:601. doi: 10.3389/fpls.2020.00601 (PMC7270330; doi:10.3389/fpls.2020.00601)
Supplement: Supplementary file 1 [file Data_Sheet_1.docx]

**
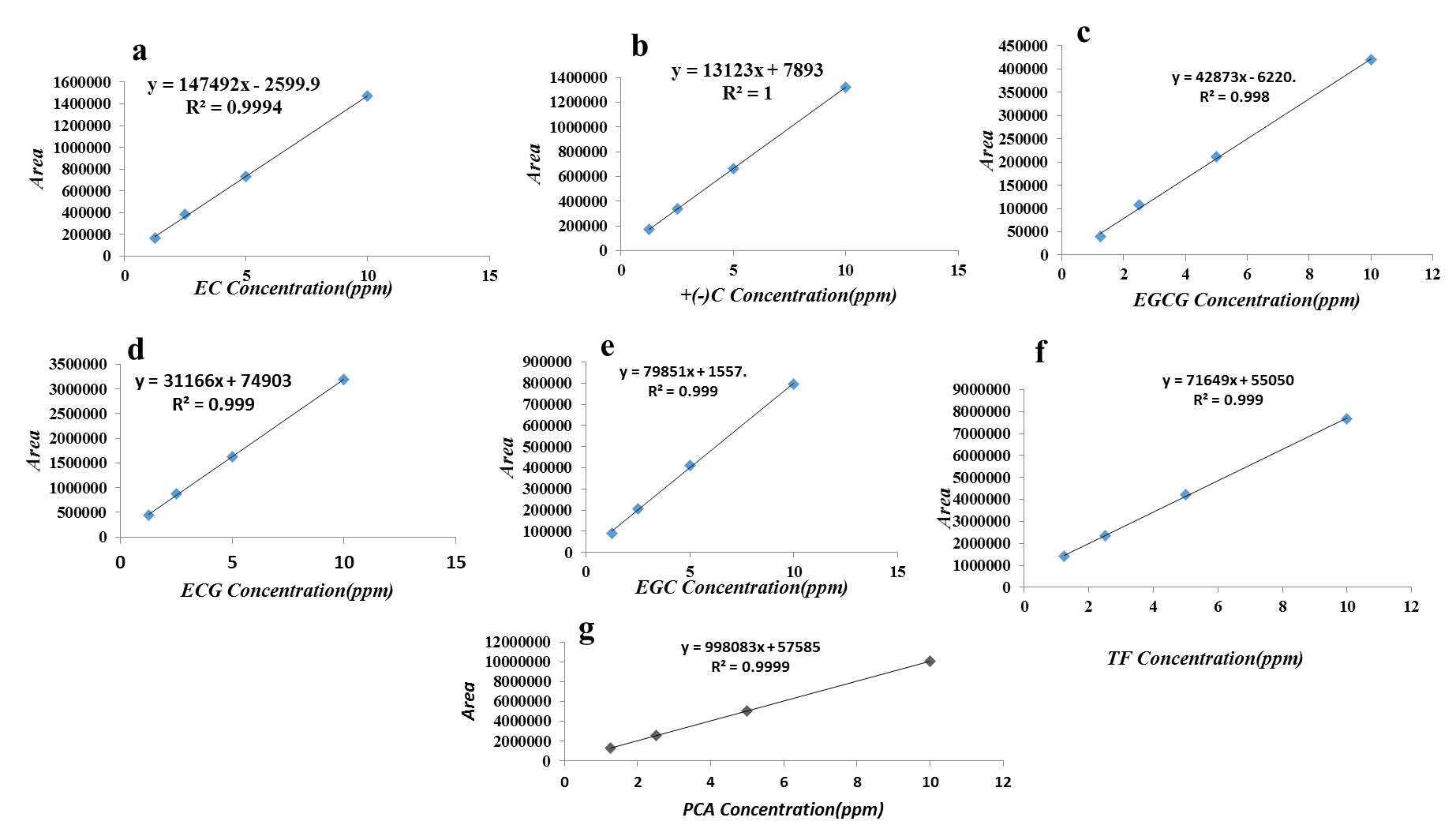
**

**Figure S1** Standard curves of epicatechin (EC), (±)catechin, epigallocatechingallate (EGCG), epicatechingallate (ECG), epigallocatechin (EGC), taxifolin (TF) , and protocatechuic acid (PCA) respectively

.


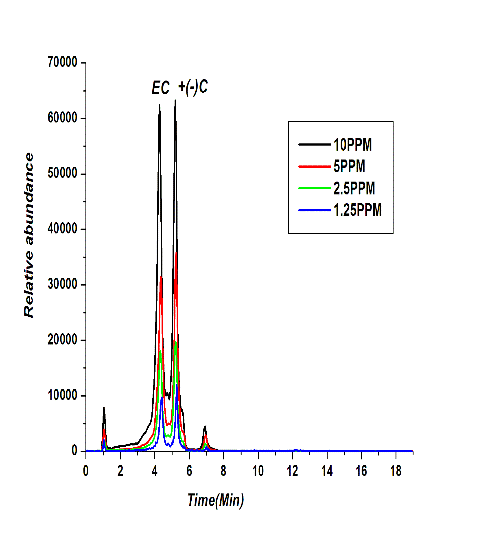

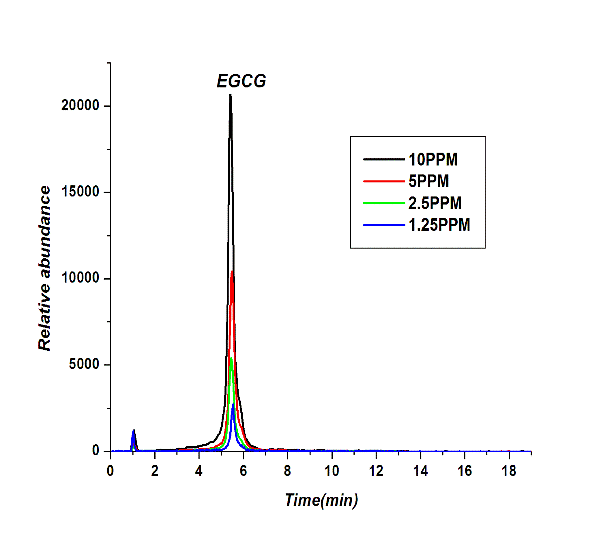

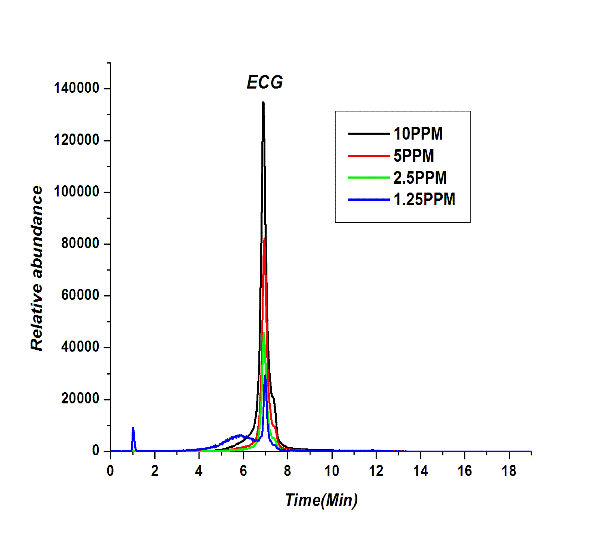

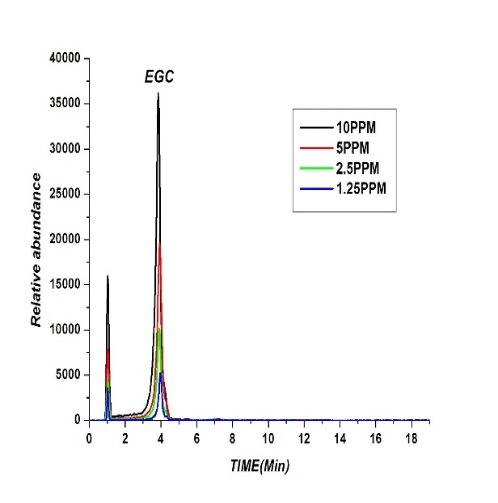

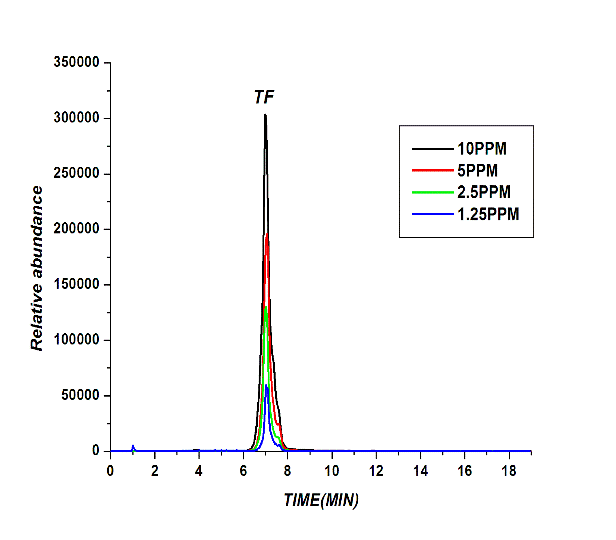

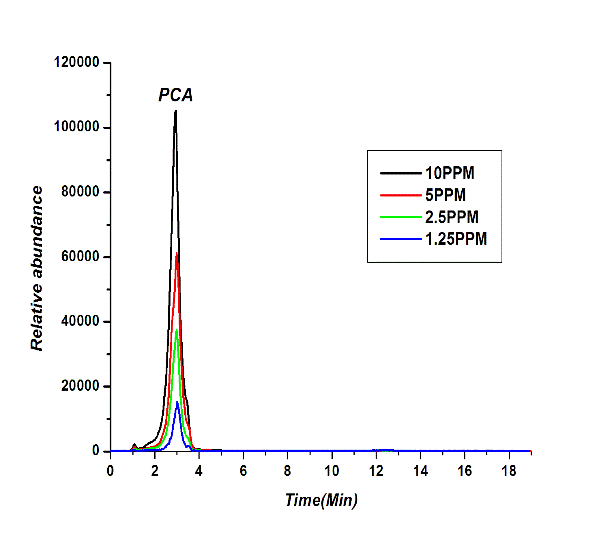


**Figure S2** Different concentrations dendograms of epicatechin (EC), (±)catechin, epigallocatechingallate (EGCG), epicatechingallate (ECG), epigallocatechin (EGC), taxifolin (TF) , and protocatechuic acid (PCA) respectively.


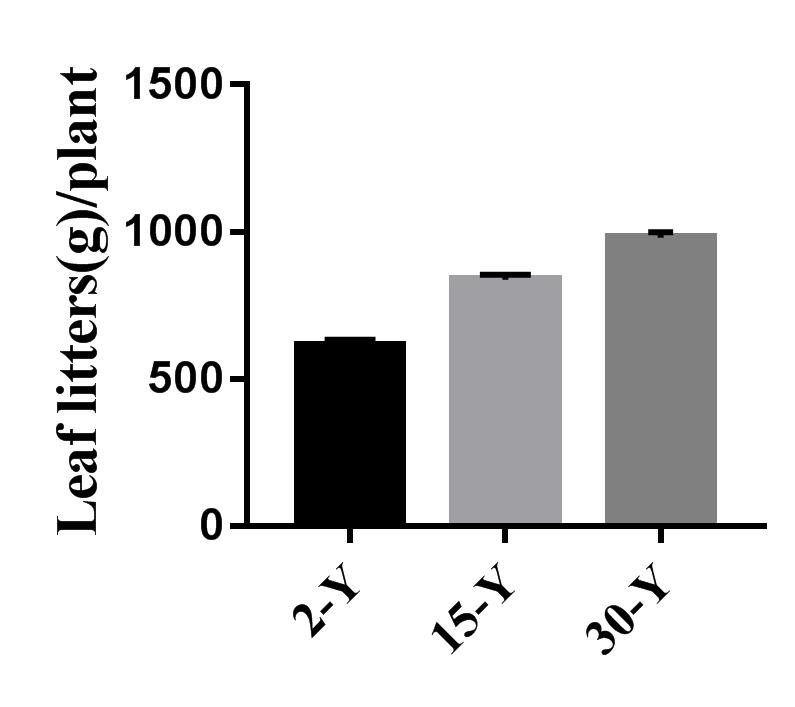


**Figure S3** Leaf letters biomass in 2 year (2-Y), 15 year (15-Y) and 30 years (30-Y) tea gardens respectively

**Table S1 Yield of different tea garden**

|  | **(Hundred-buds Weight)** | |
| --- | --- | --- |
| Treatment | (Fresh weight) | (Dry weight) |
|  | ----------g-------------- | |
| T2Y | 118.67±2.03 ^a^ | 28.87±1.55 ^a^ |
| T15Y | 92.13±2.31^b^ | 21.60±1.37 ^b^ |
| T30Y | 79.00±2.08 ^c^ | 15.95±1.34 ^c^ |

Note: 2-Y, 15-Y and 30-Y represent the newly planted 2-years’ tea field, 15 years and 30 years’ monoculture tea field respectively. Letter a and b in columns show significant difference determined by Tukey’s test (p≤ 0.05, n=100).

**Table S2 Enzyme activities of tea rhizosphere soil under different fertilizers**

| **Treatment** | **Phenol oxidase** | **Peroxidase** |
| --- | --- | --- |
|  | **--------mg·g^-1^2h^-1^--------** | |
| CK | 122.42±4.28 ^c^ | 22.03±0.52 ^c^ |
| 2-Y | 139.30±0.52 ^b^ | 24.58±0.1 ^b^ |
| 15-Y | 140.68±10.83^b^ | 24.26±0.36 ^b^ |
| 30-Y | 185.49±4.56 ^a^ | 25.97±0.22 ^a^ |

Note: CK, 2-Y, 15-Y and 30-Y represent the bulk soil, newly planted 2-years’ tea field, 15 years and 30 years’ monoculture tea field respectively. Letters a, b and c in columns show significant difference determined by Tukey’s test (p≤ 0.05, n=3).
